# Childhood family risks and mental health of older adults in China: The moderating effect of age

**DOI:** 10.3389/fpsyg.2023.994872

**Published:** 2023-02-07

**Authors:** Wang Xinzhu

**Affiliations:** Teacher Education Department, Xichang College, Xichang, China

**Keywords:** childhood family risks, mental health, older adults, moderation, age

## Abstract

**Introduction:**

Childhood family risks (CFRs) are believed to have long-arm effects on people's mental health. However, it is unclear whether age can alleviate these long-arm effects.

**Aim:**

This study aimed to explore the relationship between CFRs and the mental health of older adults (mean [*M*] = 62.10, standard deviation [SD] = 8.02) in China and investigate whether age could moderate this relationship.

**Methods:**

This cross-sectional, survey-based study used data from the Chinese General Social Survey conducted in 2015, and the data of 4,237 respondents were included in the final analysis. Mental health was measured by two items, namely negative and positive emotions. The step-by-step regression procedure and moderation analysis technique were used.

**Results:**

For older adults in China, CFRs were significantly negatively associated with their mental health (β = −0.046, *t* = −2.690, *p* < 0.01), age was positively correlated with their mental health (β = 0.115, *t* = 7.157, *p* < 0.01), and age significantly moderated the relationship between CFRs and mental health (β = 0.277, *t* = 2.172, *p* < 0.05). As age increases, the correlation between CFRs and mental health decreases, and when age is one SD above the M, CFRs are no longer significantly associated with the mental health level (*b*_simple_ = −0.01, *t* = −0.077, *p* > 0.05).

**Conclusion:**

This study showed that CFRs were negatively associated with the mental health level of older Chinese adults, and age could significantly moderate the association. Therefore, it is essential to take preventive measures in advance to protect people's mental health and teach older adults to use emotion-regulation techniques to weaken the association between CFRs and mental health.

## Introduction

Presently, most studies focus on the relationship between current factors and mental health. Regarding socio-economic factors, researchers have mostly studied income and education; and regarding individual characteristics, researchers have focused on marital status, ethnic characteristics, religious beliefs, and gender. Research has shown that compared with low-income earners, high-income earners have better mental health (Lindström and Rosvall, 2012), and people with higher education are more likely to have a better psychological state (Niemeyer et al., 2019). Moreover, people with religious beliefs are psychologically healthier than those without religious beliefs (Sharma et al., 2017; Schnabel and Schieman, 2022). Compared with single people, married people have better mental health (Lindström and Rosvall, 2012). In China, the mental health status of ethnic minorities is lower than that of the *Han* people (Hui and Hong, 2002), and women are reported to have a lower mental health level than that of men (Wei et al., 2021).

However, life is continuous, and early life events are closely related to late life developments. Early life experiences can set the stage for people's lifespan development (Hughes et al., 2017). Many scholars have regarded the early years of life as a sensitive period of development; negative experiences in this critical period of psychological development influence mental maturity in various domains (Pikhart et al., 2013). The early years of life are very crucial for cognitive, behavioral, personality, and emotional development as people develop their preferred approaches to dealing with stress and negotiating with others (Evans et al., 2013). Negative experiences in childhood—physical and psychological alike—are widely acknowledged to have long-arm effects on health long before any symptoms of illness emerge (Hughes et al., 2017; Copeland et al., 2018).

One theory that explains the association between early experiences and mental health in later life is the life course theory. This theory claims that disadvantages or adversities experienced in childhood can have enduring negative effects on later development, and the effects are observed over the entire lifespan (Yoav and Diana, 2002; Kuh et al., 2003). People are never completely separated from the imprint of their origins although their surroundings and circumstances may change (Pearlin, 2010). Drawing from the life course theory, this study explores the enduring effects of childhood family risks (CFRs) on mental health in late adulthood within the Chinese population.

A childhood risk refers to an environmental condition in a child's socialization context that could be detrimental to their normal development (Zych et al., 2021). Family is the microsystem of children's growth, and family risks are often viewed as distal risks that are consequential to one's life course (Cheung et al., 2018). Family risks can broadly be divided into the following three categories: (1) Family structure risk: It includes the death of parents, divorce of parents, and instances of remarriage; (2) Resource insufficiency risk: It refers to low socio-economic status, low parental educational attainment, and economic strain on families; and (3) Family atmosphere risk: It refers to low intimacy and frequent conflicts among family members, such as marital conflicts, ineffective parenting, and abuse (Gerard and Buehler, 2004; Pikhart et al., 2013; Chen, 2019). This study mainly focused on two risks: low socio-economic status and broken family structure.

### Childhood family risks and mental health in old age

Increasingly, several studies have examined the effects of CFRs on mental health in the later stages in China. A survey on middle school students found that accumulative CFRs can predict the students' depression and anxiety status (Xiong et al., 2020). Another study discovered that early accumulative CFRs are associated with internalization problems in Chinese adolescents (Wenming et al., 2020). Other studies have documented that college students not living with parents in childhood reported more mental diseases and suicidal ideations compared with those reported by their counterparts who lived with their parents during childhood (Liu et al., 2020, 2021). A longitudinal study demonstrates that adversities experienced in early life (e.g., parental physical abuse, maternal emotional neglect, and low parental education attainment) could exert long-term detrimental effects on the mental health of Chinese adults in their mid and late stages of life (Yang et al., 2020). Other investigations have also documented the robust association between CFRs and the mental health of older people in China (Yang and Lou, 2016; Li and Lu, 2019, 2020).

Childhood family risks can exert direct and indirect influences on health across a lifespan. Regarding direct influence, the accumulation of family risks manifests as harmful effects at three different levels—biological, behavioral, and cognitive and psychological. Biologically, childhood stress is “programmed” into macrophages endowed with pro-inflammatory tendencies, which manifest as exaggerated cytokine responses to challenges and as decreased sensitivity to inhibitory hormonal signals. The resultant inflammation drives forward pathogenic mechanisms that ultimately foster chronic diseases (Raposa et al., 2012). Behaviorally, childhood stress leads to excessive threat vigilance, poor social relationships, mistrust of others, impaired self-regulation, and unhealthy lifestyle choices (Miller et al., 2011). Cognitively and psychologically, exposure to CFRs, especially over a long period of time, may harm cognitive ability (Blair and Raver, 2016; McCoy et al., 2016), lead to suicidal ideation and major depressive disorders in later life (Hollingshaus et al., 2016), and cause many other psychotic symptoms (Trotta et al., 2015).

The indirect routes involve adversities emerging incessantly in later life. Due to the disadvantages in mental health and backward cognitive capacity caused by CFRs, individuals are less likely to fully benefit from public educational resources and other developmental opportunities, which can increase the probability of subsequent adversities in adulthood, such as low earnings, unemployment, and poorer health (Ferraro and Shippee, 2009; Schafer et al., 2013; Halonen et al., 2017). These subsequent adversities consequently lead to negative effects on physical and mental health, such as a higher likelihood of having a depressive mood and losing physical functions as well as a higher likelihood of a high mortality rate (Adda et al., 2004; Contoyannis and Jones, 2004). This logic echoes the sayings “success breeds success” and “the poor get poorer while the rich get richer.” In summary, CFRs reduce individuals' overall quality of life, exposing them to more life disadvantages in adulthood and thus affecting their physical and mental health.

### Age and emotions

Evidence has shown that age is associated with emotions (Ross and Mirowsky, 2008; Yeung et al., 2011). The socio-emotional selectivity theory (SST) posits that due to constraints on time horizons, older people tend to shift their motivational priorities such that emotional regulation becomes their most important goal (Melehin, 2015). Due to shrinking time horizons, older people develop a selective focus on positive stimuli while their attention to negative information gradually dissipates. Therefore, compared to younger people, older people are more likely to daily shift their attention from negative stimuli to positive ones and remember more positive than negative information (Carstensen, 2006). Older people are mostly present-oriented, and they dwell less on the past. Awareness of limited time in life enables them to adopt positive attitudes to buffer the impact of negative experiences and appreciate the positive aspects of their present life (Carstensen et al., 1999). A study that used the functional magnetic resonance imaging method to explore the neurobiological mechanism underlying SST found that anterior cingulate activation is related to a positivity bias and emotional stability for older populations. When processing emotional information, frontal engagement enables older people to focus on positive information and maintain a stable emotional state (Brassen et al., 2011).

Emotion regulation theory maintains that the strategies of emotional regulation improve with aging (Urry and Gross, 2010). Many studies have shown that older people deal better with emotional problems and life hassles by effectively using a wide array of problem-solving techniques, which can prevent them from the harmful impacts of negative memories and current stressful events (Blanchard-Fields, 2007). Compared with younger people, older people are more likely to positively evaluate their emotion regulation skills and focus on controlling their emotions (Mather and Carstensen, 2005). For example, when facing a difficult interpersonal situation, older people are less likely to exhibit destructive behaviors such as shouting and insulting (Birditt and Fingerman, 2005). A study found that aging was indirectly linked with emotional experience as older people used more adaptive strategies (e.g., positive reappraisal and savoring) and less maladaptive strategies (e.g., rumination and fault finding) (Scheibe et al., 2016).

### The moderation role of age

Based on the above discussion, we made two conclusions: (1) family risks tend to accumulate and have prominent and enduring effects on mental health in older stages of life, and (2) aging enables older adults to focus more on positive emotions and effectively regulate negative emotions. A question worthy of investigation arose: can aging offset the “long-arm effect” (Latham, 2015) of CFRs on mental health? In other words, can the cumulative effects of CFRs on mental health decrease with aging? Conclusions from previous studies are controversial. A study that explored the relationship between childhood adversity and psychosomatic symptoms in young adults found that the consequences of child abuse neither waned nor became substantial over time (Schafer and Ferraro, 2013). However, based on a sample of older Chinese adults, a study posited that the threat of childhood adversities to healthy aging is greatly attenuated among older people, especially for people aged above 80 years (Hu, 2021). Two scholars suggested that misfortunes in childhood have effects on health in an inverted *U* form in the process of aging. Before age 60, the harmful effects on health tend to increase; however, after 60 years, these effects begin to decrease (Lei and Ming, 2018). To the best of the author's knowledge, no studies have heretofore addressed the moderating role of age between CFRs and mental health in the Chinese population. Further research should examine the moderation effect of age more accurately.

### The aims of the present study

The current study had two objectives. First, we explored the relationship between CFRs and the mental health of the older adult population in China. Presently, China has the largest aging population in the world and has taken measures to build a positive aging society, and the mental health of the aging population has become a major concern. To help the older population maintain good mental health, it is necessary to conduct studies on the determinants of mental health, especially based on the life course theory. We proposed two hypotheses based on the earlier discussion.

Hypothesis 1: CFRs are negatively associated with older adults' level of mental health.

Second, we investigated whether age can offset the long-arm effects of CFRs on the mental health of older adults. There is a wide consensus that older people, compared with young people, are more likely to have positive emotions. However, no evidence exists to prove whether older age can weaken the long-arm negative effects of emotions. Based on the literature on aging and emotions and the study by Hu (2021), we proposed the second hypothesis as follows:

Hypothesis 2: The negative effects of CFRs on mental health will reduce with aging.

## Methods

### Data

The data used for this study was from the Chinese General Social Survey conducted in 2015 (CGSS 2015). The CGSS 2015 is a national survey that uses a stratified random sampling technique to ensure that respondents are not interviewed repeatedly, to avoid selection bias, and to ensure that the sample is representative of the whole population. The CGSS usually collects data on people's attitudes regarding current society and the relationship between social changes and quality of life in China. The CGSS 2015 provides information about citizens such as demographic characteristics, life values, and attitudes and views regarding society, employment, and income. Furthermore, the CGSS asked some questions related to respondents' CFRs, such as the characteristics of parents, and the subjective socio-economic status of their families, among others. The whole survey was conducted face-to-face, which ensured the authenticity of the information gathered. The CGSS 2015 spanned 28 provinces, including the most developed direct-controlled municipalities, Tibet Autonomous Region, and Xinjiang Autonomous Region. A total of 11,103 responses were obtained from the CGSS 2015.

According to the purpose of this study, we selected respondents aged above 50 years as the research participants. We included a total of 4,237 respondents in the final analysis after excluding respondents with incomplete information on key variables. There were 2,164 women (51.07%) and 2,073 men (48.93%), 11.90% (504) of the sample had religious beliefs and 88.10% (3,733) had no religious beliefs, and 20.30% (860) of them lived without a partner of the opposite sex and 79.70% (3,377) lived with a partner of the opposite sex. The ethnic minority constituted 6.73% (285) and the *Han* nationality constituted 93.27% (3,752) of the sample.

### Childhood family risks

This study focused mainly on low socio-economic status and broken family structure as sources of CFRs. Childhood refers to the first 14 years of life. Parental education attainment is a robust predictor of the socioeconomic grades of families (Gerard and Buehler, 2004; Wang and Luo, 2019). In China, the Communist Party membership is an important political and social capital. It provides a family with a high income, more educational opportunities, and employment opportunities (Lie et al., 2018). Parents' party membership can greatly improve the socioeconomic status of families (Mclaughlin, 2017). Previous literature has documented that self-reported social status is associated with a wide array of psychological symptoms (Moor et al., 2017; Rietz and Kuja-Halkola, 2017). There are often two forms of the broken family structure presented in the literature, namely parental divorce and parental death. The death of parents breaks the family structure and seriously affects children's psychological development (Hollingshaus et al., 2016; Appel et al., 2019).

We categorized the following events as family risks: (1) death of parents; (2) low parental education attainment (specifically, when one parent had only primary school education or below); (3) parental non-membership of the Communist Party; and (4) low self-rated family social status (specifically, when the self-rated status of a family is 3 and below on a scale of 1–10). In total, seven CFRs were obtained, and according to the studies by Schafer and Ferraro (2012) and Brimblecombe et al. (2018), we assigned the value of 1 to each CFR and summed up the scores to measure the cumulative CFRs experienced by respondents.

### Control variables

Among the current factors associated with mental health, researchers have often focused on socio-economic status including income, education, and personal characteristics, such as religion, marital status, ethnic minority, and gender. Therefore, these factors were controlled for in this study. Income in the year before the survey and respondents' educational attainment were controlled for. The income variable began from 1 to eliminate an income of 0 *yuan*. Income was subsequently transformed into natural logarithms. The education variable was continuous (no formal schooling = 0, primary schooling = 6, junior high schooling = 9, senior high schooling = 12, post-secondary education = 15, college education = 16, and post-graduate education and above = 19). The socio-demographic factors controlled for included age (number of years), gender (female = 1 and male = 0), living status (living with a partner of the opposite sex = 1 and no partner of the opposite sex = 0), nationality (ethnic minority = 0 and *Han* = 1), and religion (having religious belief = 0 and no religious belief = 1). Thus, income, education, and age were the continuous variables, and the other four were categorical variables.

### Mental health indicators

In the CGSS 2015, two items were used to assess the mental health of Chinese older adults. One item asked the respondents how often in the last month they felt depressed or dispirited, and the respondents were supposed to choose from five integers (from 1 to 5 representing always, often, sometimes, seldom, and never, respectively). The other item asked the respondents how happy they felt generally, and they were to choose from five integers (from 1 to 5 representing very unhappy, relatively unhappy, it is hard to respond with happy or not happy, relatively happy, and very happy, respectively). These two items involved both positive and negative emotion for mental health; hence, reliable measurements of mental health could be obtained (Huppert, 2010). The two items produced a high Cronbach's α index of 0.76, and their average was considered the level of mental health; higher values indicated better mental health outcomes.

### Statistical analysis

First, we used descriptive and Pearson's correlation analyses to investigate the basic characteristics and correlations among the continuous variables including age, education, income, CFRs, and mental health. Then we performed a hierarchical regression analysis to explore the relationship between CFRs and mental health and the moderation effect of age on the relationship. Specifically, we followed three steps in the linear regression analysis. In the first step, five controlling variables, including gender, living status, nationality, religion, education attainment, and income were entered into the regression model to analyze the basic effects of these variables on the dependent variable, which was mental health. In the second step, CFRs, representing the independent variable, and age were entered into the model to investigate the effect of CFRs on mental health. In step 3, the product of the interaction between age and CFRs was added to the regression model to determine how age moderated the effect of CFRs on mental health. Finally, we used the simple slope method to visually present the moderation role of age.

## Results

### Descriptive and correlation results

Descriptive statistics and correlations used to analyze the continuous variables are shown in Table 1. The scores for mental health ranged from 1 to 5, with a mean (M) score of 3.80 and a standard deviation (SD) of 0.73. The average CFR score was 4.09 (SD = 1.22), ranging from 0 to 7. Pearson's correlation analyses showed that mental health was significantly and negatively correlated with CFRs with a coefficient of −0.119 (*p* < 0.01) and significantly correlated with age with a coefficient of 0.043 (*p* < 0.01). Both income and education positively correlated with mental health.

**Table 1:** Descriptive statistics and correlations for main variables (*N* = 4,237).

		***M***	**SD**	**1**	**2**	**3**	**4**	**5**
1	Mental health	3.80	0.73	1				
2	CFRs	4.09	1.22	−0.119^**^	1			
3	Age	62.10	8.02	0.043^**^	0.162^**^	1		
4	Income (Ln)	8.05	3.50	0.177^**^	−0.229^**^	−0.081^**^	1	
5	Education	7.04	4.57	0.171^**^	−0.452^**^	−0.227^**^	0.357^**^	1

^*^p < 0.05. ^**^p < 0.01.

### Cumulative CFR and mental health

Different levels of CFRs and their corresponding mental health scores are presented in Table 2.

**Table 2:** Cumulative childhood family risks (CFRs) and mean mental health.

**CFRs**	***N***	***M***	**SD**
1 risk and below	170	3.96	0.67
2 risks	325	3.94	0.69
3 risks	617	3.92	0.68
4 risks	1,094	3.83	0.74
5 risks	1,849	3.72	0.74
6 risks	136	3.76	0.71
7 risks	46	3.55	0.63

M and SD refer to mental health values.

**Figure 1:**
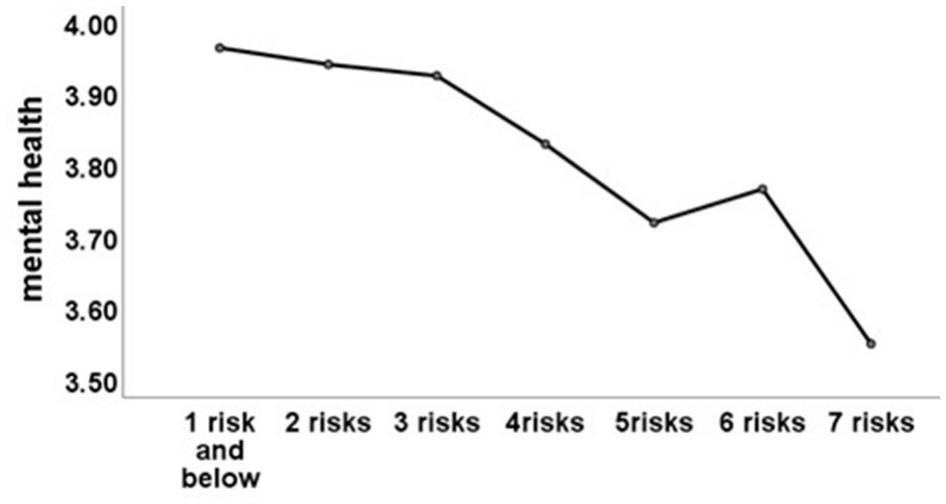
Mental health and number of childhood family risks.

Regarding the accumulation of CFRs, we found that the level of mental health for older adults decreased gradually from an average of 3.96 to 3.55, which implied that more CFRs increased the harmful effects on mental health in older age. Figure 1 clearly depicts the cumulative effect of CFRs on mental health.

### Regression analysis

The linear regression results are presented in Table 3. In step 1, mental health was considered a dependent variable. Regression results showed that gender was not significantly correlated with mental health (β = −0.005, *p* > 0.05). The other four controlling variables were each associated with mental health. Higher levels of mental health were found among *Han* nationality participants (β = 0.038, *p* < 0.05), religious people (β = −0.044, *p* < 0.01), higher income earners (β = 0.129, *p* < 0.01), highly educated people (β = 0.112, *p* < 0.01), and respondents living with a partner of the opposite sex (β = 0.047, *p* < 0.01).

**Table 3:** Linear regression results of the effect on mental health.

		**Step 1**		**Step 2**		**Step 3**	
	β	***t***	β	***t***	β	***t***
(Constant)		46.712^**^		22.166^**^		11.985^**^
Gender	−0.005	−0.290	0.000	−0.003	0.001	0.088
Living status	0.047	3.069^**^	0.077	4.838^**^	0.079	4.982^**^
Nationality	0.038	2.447^*^	0.035	2.266^**^	0.035	2.266^*^
Religion	−0.044	−2.828^**^	−0.044	−2.822^*^	−0.044	−2.874^**^
Income (Ln)	0.129	7.910^**^	0.125	7.691^**^	0.126	7.744^**^
Education	0.112	6.722^**^	0.118	6.413^**^	0.121	6.543^**^
CFRs			−0.046	−2.690^**^	−0.275	−2.573^*^
Age			0.115	7.157^**^	0.007	0.001
Age^*^ CFRs					0.277	2.172^*^
*R* ^2^	0.045		0.057		0.058	
*F*	33.928^**^		32.848^**^		29.748^**^	

^*^p < 0.05. ^**^p < 0.01. The reference groups are male, living without an opposite-sex partner, ethnic minority, and having a religious belief, respectively.

In step 2, CFRs and age were entered into the regression model, and the results showed that CFRs could significantly and negatively predict one's level of mental health (β = −0.046, *p* < 0.01), and age could significantly and positively predict mental health (β = 0.115, *p* < 0.01).

In step 3, the interaction of age and CFRs was included in the regression model, and it produced a significant prediction effect on mental health (β = 0.277, *p* < 0.05). CFRs maintained a significant effect on mental health (β = −0.275, *p* < 0.01). Furthermore, similar to the results in step 1, gender failed to produce a significant effect on mental health (β = −0.005, *p* > 0.05) and living status (β = 0.079, *p* < 0.01), whereas nationality (β = 0.035, *p* < 0.05), religion (β = −0.044, *p* < 0.01), income (β = 0.126, *p* < 0.01), and education (β = 0.121, *p* < 0.01) had significant effects on mental health.

In addition, in the second step of the regression analyses, age showed a significant prediction effect on health. However, when the product of age and CFRs was entered into the model in the third step, age failed to significantly predict mental health (β = 0.001, *p* > 0.05), indicating the prominent role of the interaction effect between age and CFRs on mental health.

### Further analysis of the moderation effect of age

**Figure 2:**
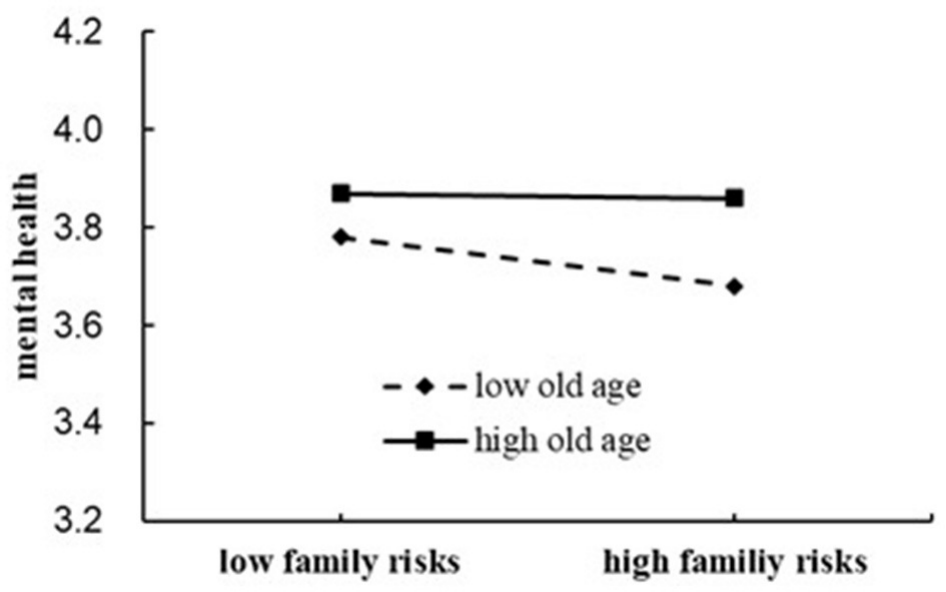
The moderating role of age.

To explore the moderation effect of age in the relationship between CFRs and mental health, we followed the procedure recommended by Hayes and Rockwood (2017) and drew a figure with simple slopes to visually show this moderation effect (see Figure 2).

When age was one SD below the M, CFRs could negatively and significantly predict mental health (*b*_simple_ = −0.037, *t* = 2.90, *p* < 0.01). However, when age increased, the cumulative impact of CFRs on mental health decreased gradually. When age was one SD above the M, CFRs could not significantly predict mental health (*b*_simple_ = −0.01, *t* = −0.077, *p* > 0.05). Therefore, we concluded that age can moderate the effect of CFRs on mental health. Thus, aging can protect older adults from the negative effect of CFRs on mental health.

## Discussion

The current study explored the relationship between CFRs and mental health in Chinese older adults. The statistical analysis supported the first hypothesis that CFRs can have negative and significant effects on mental health in old age. This finding is in agreement with previous literature. Some scholars believe that the stress experienced in childhood could exert an influence on health in one's lifespan through biological embedding or programming mechanisms, which could lead to vulnerability to stressful events or negative stimuli in later life (Miller et al., 2011). Therefore, the “long-arm effect” of CFRs in early life cannot be overlooked by improving adults' socioeconomic status. In this study, the linear regressions showed significant “long-arm effects” on mental health even when education and income were controlled for (see Table 3). The implication is that older adults' mental health is determined by factors such as individual characteristics and socioeconomic status as well as negative childhood experiences. Psychological development during various life stages is linked with each other *via* early life risk experiences, and the harmful impact of negative stress in childhood can endure through adulthood into old age. Hence, it is crucial but inadequate to target individual and psychological characteristics as well as negative childhood experiences to improve the mental health of older adults; it is necessary to address socio-economic inequality among children, especially inequalities arising from CFRs.

The second hypothesis was substantially supported as well. The linear regression of step 3 showed that the product of age and CFRs had a significant effect on mental health (see Table 3), and the simple-slope analysis further showed that the cumulative impact of CFRs could negatively and significantly predict mental health in younger adults but not significantly in older adults. Thus, the detrimental effect of CFRs on mental health is reduced with age, especially for adults above 70 years. This finding is similar to that of Lei and Ming (2018) and Hu (2021), who indicated that the threat of childhood adversities to mental health attenuated with age.

There were three main reasons to show that age could reduce the negative effects of family risks on mental health in old age. First, according to SST, because older people are aware of their limited time alive, they tend to focus more on experiences that evoke positive emotions and selectively neglect those that produce negative emotions. On the other hand, according to the emotion regulation theory, when entering old age, people use many skills to manage their emotions and maintain a positive emotional state. Third, after entering old age, people begin to pay attention to inner peace rather than external goals, focus more on their current family ties, and try to enjoy their current life (Carstensen, 2006; Ness et al., 2014). Positive emotion is the main component of mental health, so older adults can avoid the long-arm effects of CFRs on their mental health.

In addition to the hypotheses, we found that a dose-response relationship exists between CFRs and mental health in old age; that is, increasing CFRs can have more profound effects on older adults' mental health (see Table 2 and Figure 1). This finding is evidenced by other studies (Hughes et al., 2017; Felitti et al., 2019; Hu, 2021). Based on the direct pathway, accumulative CFRs can have a more profound effect on mental health in later life (Ford et al., 2011; Stalgaitis et al., 2020), and *via* the indirect pathway, low cognition capacity and health status caused by CFRs often lead to low socioeconomic status and other adversities for adults, which in turn result in a cumulative impact on mental health in old stages (Schafer et al., 2013; Lei and Ming, 2018). Therefore, the accumulation of CFRs and the adversities caused by CFRs in adulthood jointly result in a dose-response association between CFRs and mental health in the old stages.

Finally, four controlling variables, indicating the present living conditions of older adults, could significantly predict older adults' level of mental health. Living with a partner of the opposite sex, having a religious belief, belonging to the *Han* nationality, having higher education, and earning a better income are all factors that are linked with better mental health for Chinese older adults. Such results are consistent with previous research results (Hui and Hong, 2002; Lindström and Rosvall, 2012; Sharma et al., 2017; Niemeyer et al., 2019; Schnabel and Schieman, 2022). Although this study focused on early adversities rather than current factors, the relationship between current factors and mental health also needs attention.

There are some other limitations to this study in addition to the small effect sizes. First, data collection in the survey was done *via* self-reported questionnaires, thus memory bias could not be avoided in the interviewing process. Particularly, respondents, who are a group of older adults above 50 years, were required to recall information about their families during the first 14 years of life. Therefore, the accuracy and reliability of statistical analysis are likely to have been affected (Davidson et al., 2010). Second, this study contained a very limited measure for CFRs. There are many other types of CFRs according to previous literature, such as ineffective parenting and marital conflicts. The simplicity of the measure limited the findings. Third, this study used cross-sectional data. Thus, only correlational links could be inferred rather than causality links. Using longitudinal data might allow for more extensive analyses of the long-arm effects of CFRs on mental health.

## Conclusion

This study investigated the relationship between CFRs and mental health in older adulthood, using a nationally representative and large sample. Our study showed that for older adult populations, mental health was negatively associated with CFRs. Thus, the detrimental effects of CFRs on mental health are heightened with increasing levels of CFRs. On the contrary, this study found that age could moderate the effects of CFRs on mental health, and specifically, the negative effect of CFRs on mental health tended to taper in old age. Despite its limitations, the current study contributed to the knowledge regarding the long-arm effects of CFRs on mental health and the protective role of age on the mental health of Chinese older adults. Based on the findings, it is essential to take preventive measures in advance to protect people's mental health. We also propose emotion regulation-based education for adults to enhance their positive emotions and thus offset the negative effects of CFRs on mental health.

## Data availability statement

The raw data supporting the conclusions of this article will be made available by the authors, without undue reservation.

## Author contributions

The author confirms being the sole contributor of this work and has approved it for publication.
